# Development of New Mental and Physical Health Sequelae among US Veterans after COVID-19

**DOI:** 10.3390/jcm11123390

**Published:** 2022-06-13

**Authors:** Nilang Patel, Bassam Dahman, Jasmohan S. Bajaj

**Affiliations:** 1 Department of Medicine, Virginia Commonwealth University, Richmond, VA 23298, USA; jasmohan.bajaj@vcuhealth.org; 2 Division of Nephrology, Central Virginia VA Health Care System, 1201 Broad Rock Boulevard, Richmond, VA 23249, USA; 3 Department of Health Behavior and Policy, Virginia Commonwealth University, Richmond, VA 23298, USA; bassam.dahman@vcuhealth.org; 4 Department of Biostatistics, Virginia Commonwealth University, Richmond, VA 23298, USA; 5 Senior Health and Policy Analyst (WOC), Central Virginia VA Health Care System, Richmond, VA 23249, USA; 6 Division of Gastroenterology and Hepatology, Central Virginia VA Health Care System, Richmond, VA 23249, USA

**Keywords:** COVID-19, mental sequelae, physical sequelae, veterans

## Abstract

**Background:**COVID-19 sequelae among veterans need evaluation. **Design:** Propensity-score-matched retrospective cohort study. **Participants:** A total 778,738 veterans, who were tested for COVID-19 at VA facilities between 20 February 2020–27 March 2021. **Main Outcomes:** Development of new physical and mental health conditions (incidence) during the follow-up period of 7 days to 3 months after the diagnosis of COVID-19. **Results:** Out of 778,738 veterans, 149,205 (19.2%) were inpatients and 629,533 (80.8%) were outpatients. 123,757 (15.9%) diagnosed with COVID-19. Mean age was 61 ± 15.4, mostly men (89%) who were White (68%) and non-Hispanic (88%). In hospitalized patients, COVID-19 is associated with significantly higher incidences of physical conditions (venous thromboembolism (5.8% vs. 2.9%, *p* < 0.001), pulmonary circulation disorder (5.1% vs. 2.9%, *p* < 0.001), chronic lung disease (8.4% vs. 4.3%, *p* < 0.001), acute kidney injury (16.4% vs. 9.3%, *p* < 0.001), chronic kidney disease (6.5% vs. 4.8%, *p* < 0.001), cardiac arrhythmia (15.2% vs. 10.9%, *p* < 0.001), complicated hypertension (12% vs. 8.5%, *p* < 0.001), coagulopathy (6.1% vs. 2.6%, *p* < 0.001), fluid/electrolyte disorders (24.4% vs. 12.6%, *p* < 0.001) and neurological disorders (7.1% vs. 3.8%, *p* < 0.001)) and mental health conditions (depressive episode (6.6% vs. 4.3%, *p* < 0.001), adjustment disorder (2.5% vs. 1.7%, *p* < 0.001), insomnia (4.9% vs. 3.2%, *p* < 0.001) and dementia (3.0% vs. 1.9%, *p* < 0.001)) compared to propensity-matched hospitalized COVID-19 negative patients. In outpatient settings, COVID-19 diagnosis is associated with smaller increase in the incidences of the physical sequelae. **Conclusions:** In this propensity-score-matched analysis of US veterans, COVID-19 survivors, especially those who were hospitalized, developed new physical and mental health sequelae at a significantly higher rate than those without COVID-19.

## 1. Introduction

Severe acute respiratory syndrome coronavirus 2 (SARS-CoV-2) infection presents with a wide clinical spectrum ranging from asymptomatic cases to life-threatening illness. During the early part of the Coronavirus disease 2019 (COVID-19) pandemic, the emphasis was on life-threatening health consequences like severe respiratory failure, cytokine storm, thromboembolism, and death. However, as the experience with the COVID-19 has grown, a greater recognition of post-acute sequelae emerged [1,2] This could be due to the persistence of the virus in several organs and the vascular endothelium [3,4,5,6] The predictors and clinical burden of this syndrome that spans mental and physical health are being recognized across many populations but need to be described among the US Veterans.

Veterans have been vaccinated from the early part of 2021, and from mid-2021 less virulent COVID-19 variants (Alpha, Beta, Gamma, Delta, Omicron, etc.) have emerged. Vaccination, less virulent strains, and better therapeutic options have positively affected the severity and mortality of COVID-19. In general, as the years passed, milder forms of COVID-19 emerged. However, the question remains as to how the infection by the initial strain of SARS-CoV-2 affected the veterans. This vulnerable group of patients tends to be older, have higher comorbidities and disabilities, and be from lower socioeconomically disadvantaged groups than the general US population [7,8,9]. Given the larger disease burden in veterans at baseline, it is important to investigate the potential consequences of COVID-19 beyond the acute illness phase. In this study, we have investigated the development of new physical and mental conditions among veterans after the initial phase of COVID-19 infection.

## 2. Method

### 2.1. Data Source

Veteran Affairs (VA) has the largest integrated health care system in the US. All healthcare data were extracted to the VA’s Corporate Data Warehouse (CDW), which is a national electronic health data repository. To facilitate COVID-19 research, VA Informatics and Computing Infrastructure (VINCI) analysts created COVID-19 Shared Data Resource, which includes analytic tables extracted from the VA’s CDW for all patients tested for SARS-CoV-2.

### 2.2. Definition of Positive or Negative COVID-19 and Index Date

Patients were defined as COVID-19-positive if they had at least one positive polymerase chain reaction test during the study period. Patients were defined as COVID-19-negative if all polymerase chain reaction tests were negative. Final adjudication of COVID-19 status was performed by the VA National Surveillance Tool: the single, authoritative data source for the determination of positive and negative cases within the Veterans Health Administration.

The index date for all analyses was defined as the date of the earliest positive test (for COVID-19-positive patients) or the date of the earliest negative test (for COVID-19-negative patients), unless the patient had been admitted to a VA hospital during the preceding 15 days, in which case the date of admission served as the index date.

### 2.3. Study Population

We identified patients who were tested for COVID-19 at VA facilities between 20 February 2020 and 27 March 2021, for any indication, and who had at least one primary care follow-up in the previous 18 months. We excluded patients who are defined as employees and others, keeping only veterans with proven established care at the VA healthcare system. We excluded patients who died within 3 months of the index date or who did not have a minimum of 3 months of follow-up after the index date.

COVID-19-positive patients who were initially admitted and then discharged were categorized as the COVID-19-positive hospitalized cohort, whereas COVID-19 positive patients who were managed as outpatients at the time of diagnosis were categorized as the COVID-19-positive outpatient cohort.

Patients who were admitted and then discharged for other health conditions during the study period with consistent COVID-19 negative tests were categorized as COVID-19-negative hospitalized cohort, whereas patients who had COVID-19 negative tests and were managed as outpatients were categorized as the COVID-19-negative outpatient cohort.

### 2.4. Data Extraction

Available data included demographics variables like age, sex, ethnicity, race, body-mass index, and comorbid conditions. We extracted baseline comorbid conditions from CDW based on ICD-10 diagnosis codes occurring in the 2 years prior to the index date from outpatient or inpatient setting. We used the Charlson Comorbidity Index (CCI) to estimate the overall burden of baseline comorbidity.

Prevalent conditions are collected for all patients. To be considered, a prevalent health condition should have been previously recorded as ICD-10 codes in either inpatient or outpatient settings any time before the index date. For an example, to define that a patient has ischemic heart disease as a prevalent health condition, he/she should have one of the listed ICD-10 codes (ischemic heart disease—I20, I21, I22, I23 or I25) on any previous inpatient or outpatient visit. Detailed list of all ICD-10 codes for all physical and mental health conditions are listed in the supplement.

Incident conditions are defined as the development of new physical and mental health conditions (ICD-10 codes) during the follow-up, in the patients who did not have those physical and mental health conditions (ICD-10 codes) as prevalent conditions before the index date. The follow-up period is defined as 7 days to 3 months after the index date. For example, if the patient did not have a depressive episode diagnosis (ICD-10 code F32) before the index date and was subsequently, during the follow-up period, diagnosed with a depressive episode based on the ICD-10 code, then it will be considered an incident condition.

### 2.5. Outcomes

The primary outcome was the development of new physical and mental health conditions (incidence) during follow-up period in the COVID-19 positive versus the COVID-19 negative patients.

We have analyzed the following four groups: (A) COVID-19-positive hospitalized patient. (B) COVID-19-negative hospitalized patients. (C) COVID-19-positive patients managed as an outpatient. (D) COVID-19-negative patients managed as an outpatient.

### 2.6. Statistical Analysis

Patient characteristics were summarized using means and standard deviations (std) for continuous variables and percentages for categorical variables, and differences were tested with *t*-tests and χ^2^ tests, respectively.

Propensity score matching: To minimize the effects of potential confounders, and to adjust for the potential bias due to the nonrandom balance of the baseline characteristics between the COVID-19-positive and -negative patients, a propensity-matched analysis was applied [10]. Propensity scores were calculated for each subject as the predicted probability of testing positive for COVID-19 using a logistic regression model, adjusting for: age, sex, race, ethnicity, BMI, smoking status, CCI at 2 years, and state of residence. Since the patient is required to have a minimum of 3 months of the follow-up period, patients with an index date later than 27 December 2020 are not included in the analysis. Predominantly all patients were unvaccinated and infected with the initial strain of SARS-CoV-2. So, we did not match for vaccination status and SARS-CoV-2 variants. Patients were matched 1:1 without replacement using a nearest-neighbor approach with caliper restrictions. The covariate balance between the full and matched samples were evaluated using the standardized mean difference (see Supplement Section SX). Outcomes were analyzed in the propensity-matched samples using χ^2^ tests and unadjusted logistic regressions.

Stratified analysis of the incidence of the different physical and mental conditions in the full sample and in the propensity-matched samples was performed to evaluate the differences between the COVID-19 positive and negative cohorts; stratified by being in the hospitalized or the outpatient cohorts. Then a comparison between the hospitalized patients and the outpatients was performed among the COVID-19-positive patients only. All analyses were limited to patients who did not have the outcome condition prior to the index date.

Incidence rates were compared in the propensity-matched samples using χ^2^ tests. The odds ratios and 95% confidence intervals of acquiring the physical or mental condition between the comparison groups were estimated using unadjusted logistic regressions, regressing the mental or physical condition on the COVID-19 diagnosis status to compare between positive and negative cases, or on the hospitalization status to compare between the outpatient and hospitalized cohorts.

As a sensitivity analysis, the follow-up period was redefined as 15 days post index date to 3 months, and the outcomes were extracted from that period. All analyses were repeated using this definition. Another sensitivity analysis was performed by matching the hospitalized to outpatient patients among the COVID-19-positive sample. The comparison in the outcomes between hospitalized and outpatient cohorts was repeated using this matched sample.

All statistical analyses were performed using SAS Enterprise Guide version 8.2 (SAS Inc., Cary, NC, USA). Two-sided *p*< 0.001 was considered statistically significant.

This study followed the Strengthening the Reporting of Observational Studies in Epidemiology (STROBE) reporting guideline. This study was approved by the institutional review board of the Central Virginia VA healthcare system. Being a retrospective cohort analysis, a waiver of the informed consent was granted.

## 3. Results

**Figure 1:**
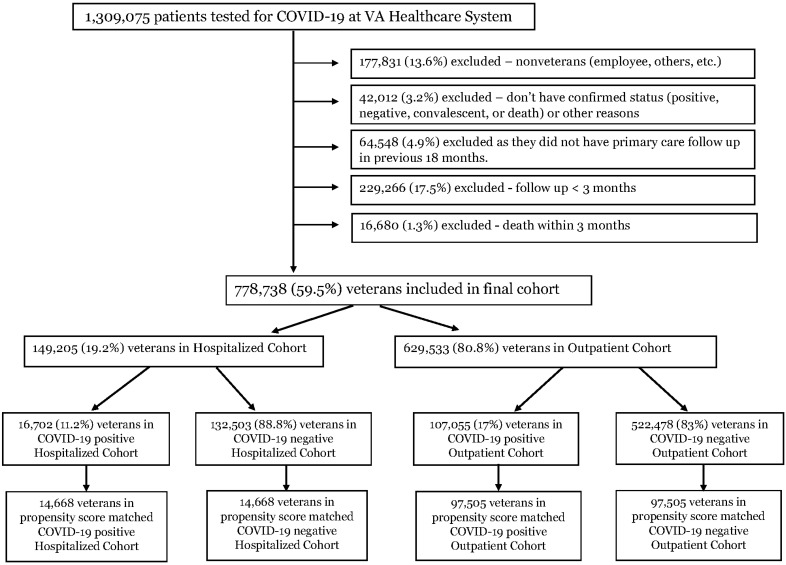
Flowchart.

Patients: We analyzed 1,309,075 patients from the VA healthcare system who were tested for COVID-19 during the study period of 20 February 2020 to 27 March 2021. After excluding various conditions as showed in Figure 1, we included 778,738 veterans for the final cohort analysis. Out of these, 149,205 (19.2%) were in the hospitalized cohort and 629,533 (80.8%) in the outpatient cohort, and 16,702 (11.2%) veterans were diagnosed with COVID-19 in the hospitalized cohort, while 107,055 (17.0%) veterans were diagnosed with COVID-19 in the outpatient cohort (Figure 1).

Mean age was 61 (±15.4). Most of the patients were male (89%), non-Hispanic ethnicity (88%), and White (68%). (Table S1). In bivariate analysis of demographic variables at baseline, Hispanic ethnicity, Black race, and higher BMI are associated with COVID-19 positivity. However, slightly higher comorbidity burden (CCI) and older age were found in COVID-19 negative patients (Table 1).

Incidence of physical and mental health conditions between COVID-19-negative and COVID-19-positive patients’ unmatched samples, stratified by the hospitalized and outpatient cohort, is reported in Table S2.

After propensity-score-matched analysis, the hospitalized COVID-19-positive and -negative groups had 14,668 patients each, and each of the outpatient COVID-19-positive and -negative groups had 97,505 patients (Figure 1).

**Figure 2:**
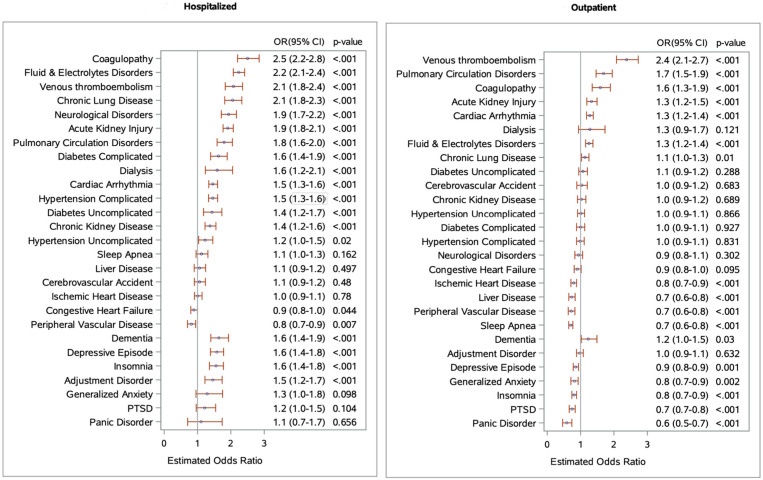
Likelihood of development of new physical and mental health conditions in COVID-19-positive patients compared to COVID-19-negative patients. The figure (**a**) represents the odds ratio for the development of new physical and mental health conditions among propensity-score-matched hospitalized COVID-19 positive patients versus hospitalized COVID-19-negative patients during the follow-up period of 7 days to 3 months of diagnosis. For example—COVID-19-positive patients are 1.4 times (OR: 1.4, 95% CI 1.2–1.6) more likely to develop chronic kidney disease than COVID-19-negative patients during the follow-up period of 7 days to 3 months of diagnosis. The figure (**b**) represents the odds ratio for the development of new physical and mental health conditions among matched outpatient COVID-19-positive patients versus outpatient COVID-19-negative patients.

Incidence of the physical and mental health conditions is reported in Table 2. Likelihoods of developing the physical and mental health conditions among COVID-19-positive patients compared to COVID-19-negative patients are reported in Figure 2.

### 3.1. COVID-19 Positive versus Negative Comparisons

Pulmonary: In the hospitalized cohort, COVID-19 diagnosis is associated with a 5.8% incidence of venous thromboembolism (VTE) compared to 2.9% in the COVID-19-negative group (*p* < 0.001). Similarly, the incidence of pulmonary circulation disorder and chronic lung disease are 5.1% and 8.4% in the COVID-19-positive group, compared to 2.9% and 4.3% in the COVID-19-negative group, respectively (*p* < 0.001). When we compared the outpatient cohort, we found that COVID-19 diagnosis is associated with a rise in the incidence of the above conditions, but the incidence rate is much smaller. (Table 2).

Renal: In the hospitalized cohort, we noted a 16.4% incidence of acute kidney injury (AKI) in the COVID-19-positive group compared to 9.3% in the COVID-19-negative group (*p* < 0.001). Interestingly, AKI incidence was also high in the COVID-19-positive outpatient cohort. Incidence of dialysis (1.1% vs. 0.69%, *p* < 0.001) and chronic kidney disease (CKD) (6.5% vs. 4.8%, *p* < 0.001) were high in the hospitalized COVID-19-positive group compared to the hospitalized COVID-19-negative group. However, they were not much different in the outpatient cohort. (Table 2).

Cardiovascular: We found a higher incidence of cardiac arrhythmias (15.2% vs. 10.9%, *p* < 0.001) and complicated hypertension (12% vs. 8.5%, *p* < 0.001) in hospitalized COVID-19-positive patients versus hospitalized COVID-19-negative patients. A higher incidence of cardiac arrhythmias (1.9% vs. 1.5%, *p* < 0.001) was also noted in the outpatient setting in the COVID-19-positive patients. Interestingly, the incidence of CHF and PVD were higher in hospitalized COVID-19-negative cohort, while the incidence of ischemic heart disease and cerebrovascular accidents (CVA) were not much different between groups. (Table 2).

Other physical conditions: COVID-19 diagnosis was also associated with higher incidence of fluid and electrolyte disorders (24.4% vs. 12.6%, *p* < 0.001), coagulopathy (6.1% vs 2.6%, *p* < 0.001), and neurological disorders (7.1% vs. 3.8%, *p* < 0.001) in the hospitalized cohort. Even with the outpatient cohort, a higher incidence of coagulopathy and fluid–electrolyte disorder were noted, but absolute values were low. (Table 2).

Mental health disorders: In the hospitalized cohort, COVID-19 was associated with a significantly higher incidence of depressive disorder (6.6% vs. 4.3%, *p* < 0.001), adjustment disorder (2.5% vs. 1.7%, *p* < 0.001), insomnia (4.9% vs. 3.2%, *p* < 0.001), and dementia (3.0% vs. 1.9%, *p* < 0.001) compared to the matched COVID-19-negative patients. There was not much difference in the outpatient cohort. (Table 2).

### 3.2. COVID-19 Positive Patients Hospitalized versus Outpatient Comparison

This comparison showed that hospitalized patients with COVID-19 had a significantly higher incidence and odds of development of physical and mental health conditions compared to those who were managed as an outpatient. (Table 3, Figure S1).

The sensitivity analysis by redefining the follow-up period as 15 days post index date to 3 months showed consistent results for all outcomes. (Tables S3 and S4).

## 4. Discussion

In a large propensity-score-matched analysis of the Veteran population we found that COVID-19 survivors have a significantly higher rate of the development of new physical and mental health sequelae. The risk of developing these sequelae increase in those who required hospitalization for COVID-19.

Veterans are predisposed to several mental health disorders stemming from war-related and other exposures. This burden is higher among US veterans compared to the general population. After critical illnesses, a subgroup of patients develops adjustment disorders or even PTSD. We found that veterans who required hospitalization for COVID-19 have higher incidence of development of new mental health disorders, mainly depressive episodes, adjustment disorder, PTSD, insomnia, and dementia, compared to matched COVID-19-negative hospitalized patients and the matched COVID-19-positive outpatient group. This increase was not trivial since it was 1.3 to 10 folds compared to the matched groups. The increase was highest in dementia, insomnia, and depressive disorder, and relatively lower for PTSD and panic episodes. These data extend other studies into the US veteran realm [1,11,12]. In a recently published French study, at 4-month follow-up telephone interviews 17.5% reported memory problems, and 20.7% reported cognitive symptoms [13] The neuroinvasive properties of SARS-CoV-2 and neuroinflammation are some of the speculative mechanisms that could explain higher neuropsychiatric manifestation in COVID-19 patients [3,4,14]. These are important consequences that need a priori goal-setting for the patient and the VHA system as a whole. Even under the usual circumstances, mental health disorders among veterans are independently associated with greater health care utilization, rates of disability, and mortality [15,16,17]. Social isolation, anxiety, fear of contagion, uncertainty, chronic stress, and the economic difficulties of the pandemic may lead to the development or exacerbation of depression, anxiety, substance use, and other psychiatric disorders among vulnerable populations [18]. Therefore, any additional increase in the incidence of mental health illnesses among veterans is a concerning finding and needs more attention to prevent long-term health consequences.

These mental health changes were accompanied by major alterations in renal, lung, cardiovascular, and thrombotic complications. As expected, lung-related complications were greater in those recovering from COVID-19 with almost double the incidence of chronic lung disease and pulmonary circulatory disorder in COVID-19 survivors. Our result is consistent with previously reported data, where 63–71% COVID-19 survivors had radiological abnormalities consistent with pulmonary dysfunction, 19% had fibrotic lesions in the lung, and 25–53% had decreased diffusion capacity for carbon monoxide [13,19,20]. These results focused on the lung complications, and validated the dataset and potentially the methodology used to determine the incidence of complications after COVID-19 in this dataset. Prior analyses of renal complications in patients post-COVID have shown a worsening eGFR trajectory, proteinuria, hematuria, and new-onset kidney failure as an outpatient [13,21]. Our analyses point towards a greater rate of AKI in hospitalized veterans with COVID-19 compared to those hospitalized without it. Since we excluded patients who died within 3 months, our AKI incidence is lower than previously reported in the literature [22]. Also, in keeping with prior literature, we found almost a doubling of VTE incidence in hospitalized COVID-19 survivors. This is likely exacerbated by endothelial dysfunction, cytokine release, and various pro-inflammatory milieux in this condition [23]. Previously reported data showed the incidence of VTEs almost up to 50%; however, it also carries a very high mortality rate [24,25]. Since we have excluded veterans who died within 3 months of having COVID-19, our reported incidence is lower. We also found a higher incidence of cardiac arrhythmia in COVID-19 survivors. Some prior uncontrolled studies that did not distinguish prevalence from incidence describe a rate of cardiac arrhythmia in COVID-19 in the range of 6–21% [26,27,28]. Medications, direct COVID-19 cardiac injury, or electrolyte disorders could be contributory [29].

We also found that the incidences of several other conditions were higher in COVID-19-negative survivors compared to those with COVID-19. Specifically, these were related to CHF and PVD. Similar behavior seen in other conditions such as cirrhosis [30], it is likely that patients seeking admission for COVID-19-unrelated issues during the pandemic are more advanced in their disease process. At baseline, US veterans have a larger comorbid condition burden than the general US population. In our cohort, this was demonstrated by the higher baseline Charleston comorbidity index in the COVID-19-negative group. Therefore, these results as a whole demonstrate that, despite propensity-matching, there is a selective increase in incidence of mental health, thromboembolic, pulmonary, and renal complications in hospitalized survivors of COVID-19 but not in the usual conditions responsible for pre-COVID-19 care in VHA, such as PVD and CHF. This adds confidence that the data is specific for COVID-19 and not simply a function of hospitalization during the pandemic in the VA system.

Our study has several strengths. We have a large study population with propensity-score-matched analysis to focus on COVID-19-related impacts, have also analyzed hospitalized to non-hospitalized COVID-19 patients, and done sensitivity analysis. Because we have relied on administrative data, our study is not subject to biases inherent in self-report studies or questionnaire-based studies of long COVID-19 outcomes.

Our study has several limitations. First, our study population is predominantly male veterans, so the result cannot be generalized to other US populations. Second, although, we have only included veterans with established primary care in the VA healthcare system, some veterans might have obtained the care outside the VA system. Third, we have only captured illness based on ICD-10 codes. Fourth, despite the propensity score-matching, there could be residual confounding factors. Fifth, since there is no consistent definition of post-acute sequelae, we were not able to determine pre-discharge variables that could determine who develops this diagnosis. Instead, we treated all new diseases as potentially related to COVID-19 and analyzed the severity of COVID-19 (hospitalized or not) as a comparator. Lastly, clinicians might have paid more attention to the patient during the follow-up visit after the COVID-19 diagnosis and that could have led to improved detection of the health conditions (e.g., dementia), which had been present but remained undiagnosed before COVID-19 diagnosis.

We conclude that in a large propensity-score-matched analysis, veterans who survived COVID-19 developed new physical and mental health comorbidities at a much higher rate compared to those who were hospitalized without COVID-19 and those with COVID-19 without hospitalization. Since, veterans have a higher comorbidity burden to begin with, any additional increase in the incidence of physical and mental health sequelae resulting from COVID-19 is a concerning finding that needs to inform policy and healthcare system changes within the VHA and beyond.

## Figures and Tables

**Table 1:** Baseline Characteristics of patients in different cohorts.

	Hospitalized Cohort					Outpatient Cohort			
	Total, *n* (%)	COVID-19-Negative, *n* (%)	COVID-19-Positive, *n* (%)	*p* Value		Total, *n* (%)	COVID-19-Negative, *n* (%)	COVID-19-Positive, *n* (%)	*p* Value
**Age, mean (±SD)**	68.0 ± 12.8	68.1 ± 12.8	67.8 ± 13.3	0.02		59.4 ± 15.5	59.5 ± 15.4	58.4 ± 16.2	<0.001
**Gender**									
Male	140,125 (93.9%)	124,371 (93.9%)	15,754 (94.3%)	0.02		550,403 (87.4%)	455,736 (87.2%)	94,667 (88.4%)	<0.001
Female	9080 (6.1%)	8132 (6.2%)	948 (5.7%)			79,130 (12.6%)	66,742 (12.8%)	12,388 (11.6%)	
**Ethnicity**									
Non-Hispanic	135,545 (90.8%)	120,639 (91.0%)	14,906 (89.2%)	<0.001		552,005 (87.7%)	460,315 (88.1%)	91,690 (85.6%)	<0.001
Hispanic	9314 (6.2%)	7963 (6.0%)	1351 (8.09%)			56,244 (9.0%)	44,698 (8.6%)	11,546 (10.8%)	
Unknown	4346 (2.9%)	3901 (2.9%)	445 (2.7%)			21,284 (3.3%)	17,465 (3.3%)	3819 (3.6%)	
**Race**									
White	103,974 (69.7%)	93,517 (70.6%)	10,457 (62.6%)	<0.001		426,534 (67.8%)	354,595 (67.9%)	71,939 (67.2%)	<0.001
Black or African American	34,237 (22.9%)	29,344 (22.1%)	4893 (29.3%)			140,655 (22.3%)	116,821 (22.4%)	23,834 (22.3%)	
American Indian or Alaska Native	1121 (0.8%)	973 (0.7%)	148 (0.9%)			5178 (0.8%)	4159 (0.8%)	1019 (1.0%)	
Asian	807 (0.5%)	687 (0.5%)	120 (0.7%)			8045 (1.3%)	6990 (1.3%)	1055 (1.0%)	
Native Hawaiian or Other Pacific Islander	1009 (0.7%)	862 (0.7%)	147 (0.9%)			5921 (0.9%)	4906 (0.9%)	1015 (1.0%)	
							
							
**BMI, mean (±SD)**	29.2 ± 6.96	29.1 ± 6.94	30.1 ± 7.04	<0.001		30.3 ± 6.23	30.1 ± 6.21	31.2 ± 6.23	<0.001
**CCI, mean (±SD)**	3.18 ± 2.73	3.18 ± 2.73	3.14 ± 2.73	0.09		1.66 ± 2.08	1.70 ± 2.11	1.48 ± 1.94	<0.001

**Table 2:** Incidence of physical and mental conditions between COVID-19-negative vs. -positive patients (Matched sample).

	Hospitalized Cohort				Outpatient Cohort		
	COVID-19 Negative, *n* (%)	COVID-19 Positive, *n* (%)	*p* Value		COVID-19 Negative, *n* (%)	COVID-19 Positive, *n* (%)	*p* Value
**Pulmonary**							
Venous Thromboembolism	384 (2.9%)	769 (5.8%)	<0.001		293 (0.3%)	696 (0.7%)	<0.001
Pulmonary Circulation Disorders	386 (2.9%)	673 (5.1%)	<0.001		299 (0.3%)	507 (0.5%)	<0.001
Sleep Apnea	306 (3.4%)	330 (3.8%)	0.16		1238 (2.1%)	906 (1.5%)	<0.001
Chronic Lung Disease	404 (4.3%)	768 (8.4%)	<0.001		777 (1.1%)	907 (1.2%)	0.01
**Renal**							
Acute Kidney Injury	1070 (9.3%)	1834 (16.4%)	<0.001		506 (0.6%)	677 (0.7%)	<0.001
Chronic Kidney Disease	510 (4.8%)	665 (6.5%)	<0.001		514 (0.6%)	523 (0.6%)	0.69
Dialysis	98 (0.7%)	154 (1.1%)	<0.001		73 (0.1%)	93 (0.1%)	0.12
**Cardiovascular**							
Ischemic Heart Disease	664 (7.5%)	679 (7.6%)	0.78		751 (1.0%)	601 (0.8%)	<0.001
Cerebrovascular Accident	321 (2.5%)	332 (2.7%)	0.48		333 (0.4%)	342 (0.4%)	0.68
Congestive Heart Failure	717 (6.6%)	644 (6.0%)	0.04		610 (0.7%)	557 (0.6%)	0.10
Peripheral Vascular Disease	386 (3.5%)	319 (2.8%)	0.007		486 (0.6%)	355 (0.4%)	<0.001
Cardiac Arrhythmia	929 (10.9%)	1317 (15.2%)	<0.001		1106 (1.5%)	1442 (1.9%)	<0.001
Hypertension Uncomplicated	270 (10.6%)	312 (12.7%)	0.02		717 (2.0%)	720 (2.0%)	0.87
Hypertension Complicated	845 (8.5%)	1168 (12.0%)	<0.0001		590 (0.7%)	585 (0.7%)	0.83
**Endocrine**							
Diabetes Uncomplicated	192 (2.4%)	249 (3.4%)	<0.001		529 (0.8%)	539 (0.8%)	0.29
Diabetes Complicated	289 (3.3%)	428 (5.2%)	<0.001		525 (0.7%)	506 (0.7%)	0.93
**Others**							
Liver Disease	294 (2.4%)	311 (2.5%)	0.50		546 (0.6%)	407 (0.5%)	<0.001
Coagulopathy	341 (2.6%)	817 (6.1%)	<0.001		219 (0.2%)	351 (0.4%)	<0.001
Fluid and Electrolytes Disorders	1229 (12.6%)	2316 (24.4%)	<0.001		912 (1.1%)	1167 (1.4%)	<0.001
Neurological Disorders	458 (3.8%)	817 (7.1%)	<0.001		512 (0.6%)	484 (0.6%)	0.30
**Mental Disorders**							
Depressive Episode	428 (4.3%)	655 (6.6%)	<0.001		979 (1.5%)	875 (1.3%)	<0.001
Panic Disorder	36 (0.3%)	40 (0.3%)	0.66		189 (0.2%)	111 (0.1%)	<0.001
Generalized Anxiety	74 (0.5%)	96 (0.7%)	0.10		481 (0.6%)	394 (0.4%)	0.002
PTSD	126 (1.1%)	151 (1.4%)	0.10		625 (1.0%)	486 (0.7%)	<0.001
Adjustment Disorders	214 (1.7%)	308 (2.5%)	<0.001		730 (0.9%)	720 (0.9%)	0.63
Insomnia	367 (3.2%)	553 (4.9%)	<0.001		1118 (1.5%)	932 (1.2%)	<0.001
Dementia	251 (1.9%)	393 (3.0%)	<0.001		197 (0.2%)	240 (0.3%)	0.03

**Table 3:** Incidence of physical and mental conditions between COVID-19-positive hospitalized vs. COVID-19-positive outpatient cohort (matched sample).

All COVID-19-Positive Patients	TOTAL, *n* (%)	Hospitalized, *n* (%)	Outpatients, *n* (%)	*p* Value
**Pulmonary**				
Venous Thromboembolism	912 (3.37%)	763 (5.74%)	149 (1.08%)	<0.001
Pulmonary Circulation Disorders	790 (2.92%)	667 (5.03%)	123 (0.892%)	<0.001
Sleep Apnea	457 (2.59%)	333 (3.83%)	124 (1.39%)	<0.001
Chronic Lung Disease	950 (5.09%)	769 (8.44%)	181 (1.89%)	<0.001
**Renal**				
Acute Kidney Injury	2013 (8.45%)	1826 (16.3%)	187 (1.48%)	<0.001
Chronic Kidney Disease	777 (3.75%)	664 (6.48%)	113 (1.08%)	<0.001
Dialysis	186 (0.661%)	154 (1.10%)	32 (0.226%)	<0.001
**Cardiovascular**				
Ischemic Heart Disease	799 (4.32%)	675 (7.56%)	124 (1.30%)	<0.001
Cerebrovascular Accident	409 (1.63%)	334 (2.69%)	75 (0.588%)	<0.001
Congestive Heart Failure	799 (3.55%)	642 (5.94%)	157 (1.34%)	<0.001
Peripheral Vascular Disease	407 (1.77%)	318 (2.82%)	89 (0.758%)	<0.001
Cardiac Arrhythmia	1560 (8.46%)	1312 (15.1%)	248 (2.53%)	<0.001
Hypertension Uncomplicated	396 (7.28%)	312 (12.6%)	84 (2.82%)	<0.001
Hypertension Complicated	1326 (6.46%)	1165 (11.9%)	161 (1.50%)	<0.001
**Endocrine**				
Diabetes Uncomplicated	333 (2.27%)	250 (3.42%)	83 (1.12%)	<0.001
Diabetes Complicated	514 (3.07%)	431 (5.21%)	83 (0.979%)	<0.001
**Others**				
Liver Disease	371 (1.50%)	310 (2.53%)	61 (0.486%)	<0.001
Coagulopathy	911 (3.37%)	811 (6.11%)	100 (.728%)	<0.001
Fluid and Electrolytes Disorders	2603 (12.5%)	2318 (24.4%)	285 (2.51%)	<0.001
Neurological Disorders	949 (3.98%)	815 (7.10%)	134 (1.09%)	<0.001
**Mental Disorders**				
Depressive Episode	783 (3.83%)	654 (6.56%)	129 (1.23%)	<0.001
Panic Disorder	56 (0.196%)	40 (0.280%)	16 (0.112%)	<0.001
Generalized Anxiety	133 (0.489%)	95 (0.698%)	38 (0.280%)	<0.001
PTSD	209 (0.953%)	151 (1.37%)	58 (0.532%)	<0.001
Adjustment Disorder	412 (1.64%)	304 (2.44%)	108 (0.855%)	<0.001
Insomnia	682 (2.97%)	550 (4.86%)	132 (1.13%)	<0.001
Dementia	465 (1.78%)	392 (3.04%)	73 (0.553%)	<0.001

## Supplementary Materials

The following supporting information can be downloaded at: https://www.mdpi.com/article/10.3390/jcm11123390/s1.
