# Supplementary material for: Development of New Mental and Physical Health Sequelae among US Veterans after COVID-19
Source: J Clin Med. 2022 Jun 13;11(12):3390. doi: 10.3390/jcm11123390 (PMC9225186; doi:10.3390/jcm11123390)
Supplement: Supplementary file 1 [file jcm-11-03390-s001.zip › jcm-1712243-supplementary.pdf]

Supplements

1). Supplement Appendix X – Propensity score matching analysis – Page 2

2). Table S1 - Baseline Characteristics of the Total Cohort - Page 5

3). Table S2 - Incidence of Physical and Mental conditions between COVID-19 Negative vs. Positive Patients (Un-matched sample) – Page 6

4). Figure S1 - Likelihood of development of new physical and mental health conditions among matched COVID-19 positive hospitalized patients compared to matched COVID-19 positive outpatient. – Page 8

5). Table S3 - Incidence of Physical and Mental conditions between matched COVID-19 Negative vs. Positive Patients (Follow up period – 15 days post-index to 3 months) – Page 9

6). Table S4 - Incidence of Physical and Mental conditions between matched hospitalized COVID-19 positive vs. outpatient COVID-19 positive. (Follow up period – 15 days post-index to 3 months) – Page 11

7). ICD-10 codes to define Physical and Mental Health Conditions - Page 12

Supplement Section SX

Distribution of Propensity Scores before Matching

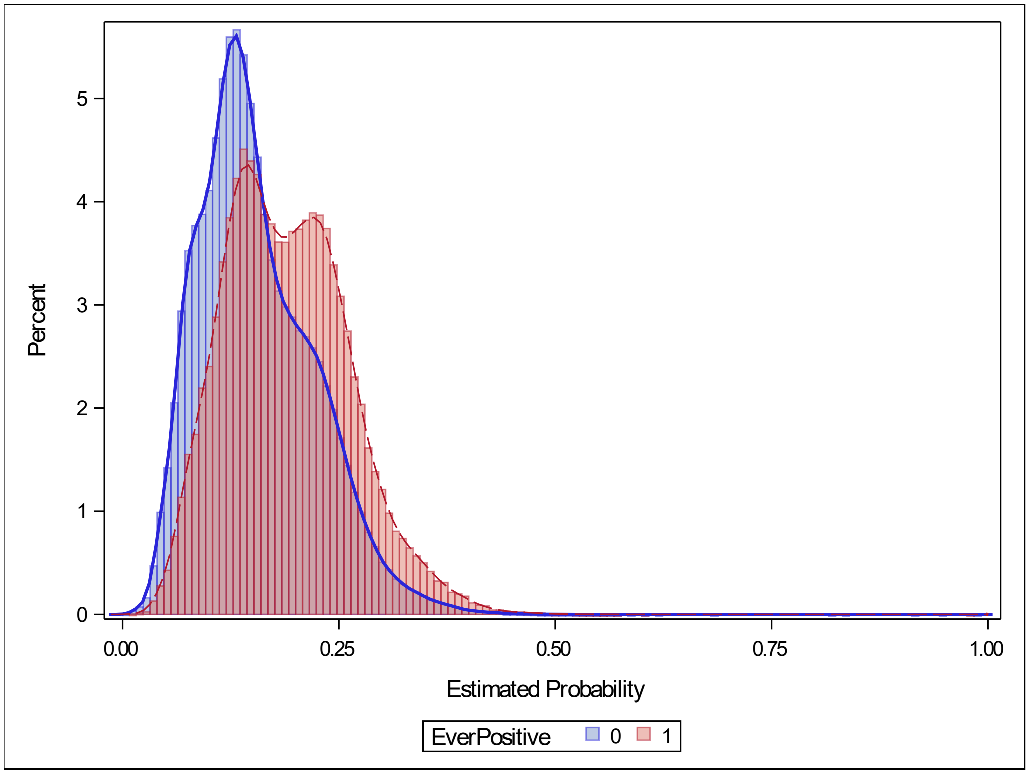

The range of extended support region of propensity scores was (0.00821, 0.980492)

Total number of

	Sample Size	Within Support Region	Matched
COVID19 Positive	112,373	112,373	112,373
COVID19 Negative	586,183	586,150	112,373

Balance of Baseline characteristics before and after matching: Love Plot

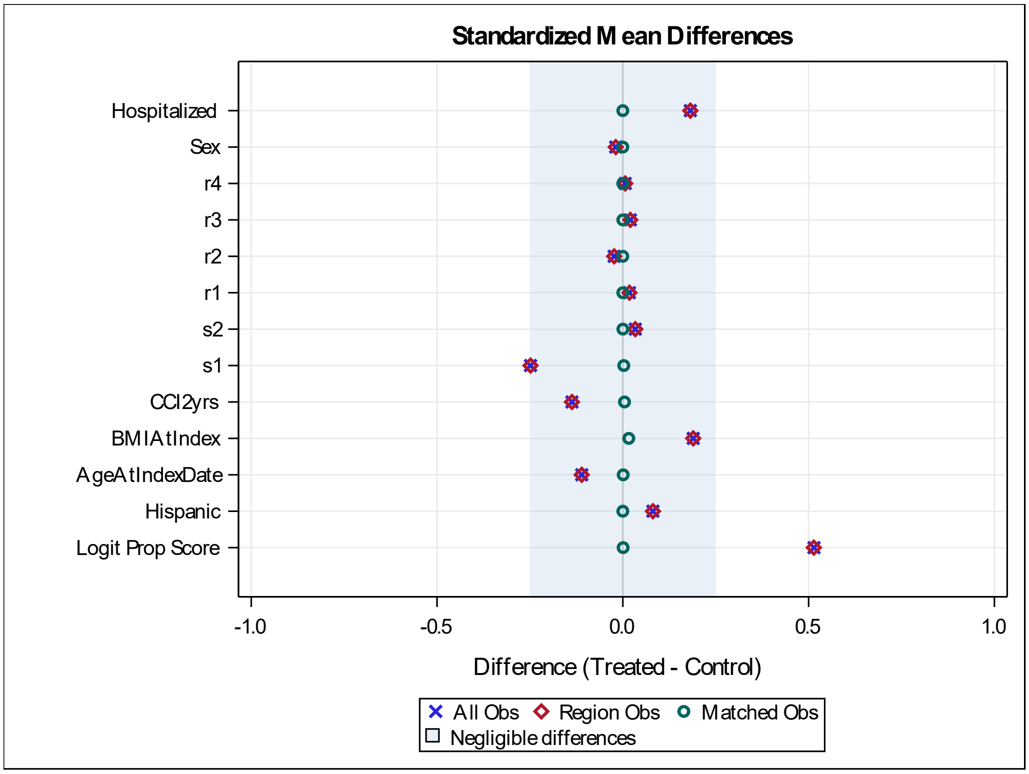

s1: Current smoker, s2: Former smoker,. r1: American Indian or Alaskan Native, r2: Asian, r3: Black or African American, r4: Native Hawaiian or Other Pacific Islander, r5: Unknown race, r6: White.

Distribution of Propensity Scores After Matching

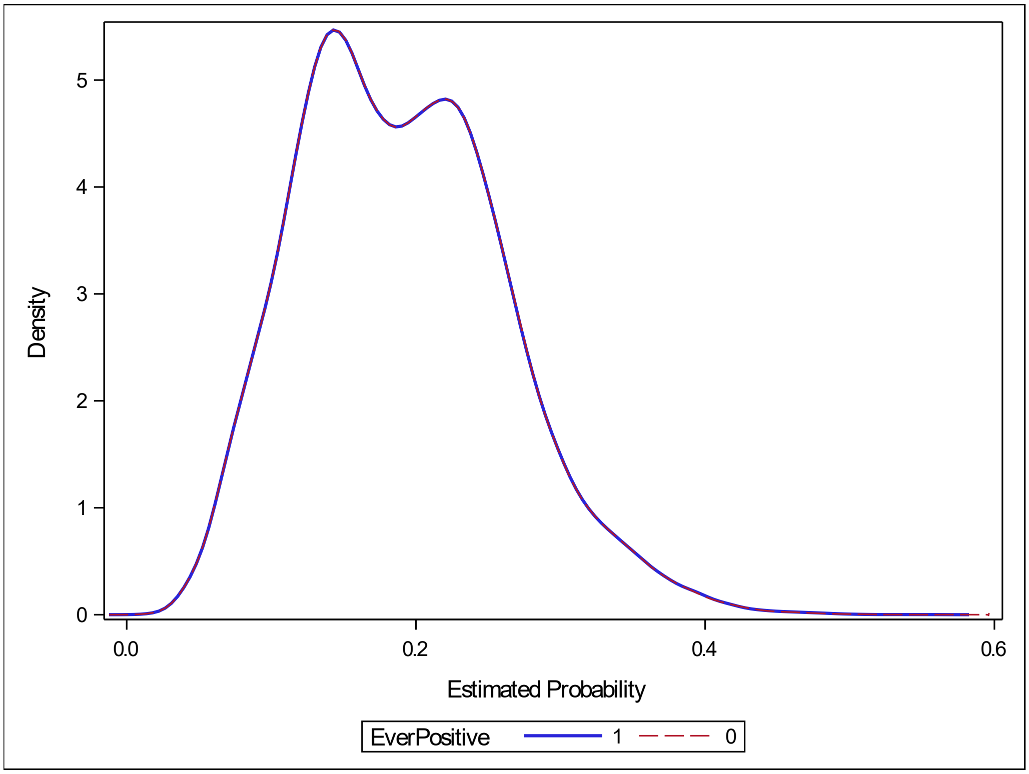

Standardized Mean Differences between COVID19 Positive and COVID19 Negative Cases

Variable	Observations	Mean Difference	Standard Deviation	Standardized Difference	Percent Reduction	Variance Ratio
Logit Prop Score	All	0.266	0.518	0.514		0.87
	Matched	0.000		0.001	99.85	1.00
Hispanic	All	0.023	0.287	0.081		1.26
	Matched	0		0	100	1.00
Age At Index Date	All	-1.709	15.459	-0.111		1.12
	Matched	0.015		0.001	99.13	1.01
BMI At Index Date	All	1.204	6.355	0.189		1.00
	Matched	0.102		0.016	91.5	0.92
Charlson Comorbidity Index (past 2 years)	All	-0.306	2.231	-0.137		0.84
	Matched	0.010		0.005	96.69	1.02
s1	All	-0.092	0.370	-0.248		0.63
	Matched	0.001		0.003	98.96	1.01
s2	All	0.017	0.495	0.034		1.01
	Matched	0.000		0.000	99.36	1.00
r1	All	0.002	0.092	0.018		1.21
	Matched	0		0	100	1.00
r2	All	-0.002	0.103	-0.023		0.80
	Matched	0		0	100	1.00
r3	All	0.008	0.420	0.020		1.03
	Matched	0		0	100	1.00
r4	All	0.001	0.096	0.006		1.07
	Matched	0		0	100	1.00
Male	All	-0.006	0.312	-0.019		0.95
	Matched	0		0	100	1.00
Hospitalized	All	0.067	0.369	0.182		0.72
	Matched	0		0	100	1.00

Standard deviation of All observations used to compute standardized differences. s1: Current smoker, s2: Former smoker, s3: Never smoker, S4 missing smoking history. r1: American Indian or Alaskan Native, r2: Asian, r3: Black or African American, r4: Native Hawaiian or Other Pacific Islander, r5: Unknown race, r6: White.

Table S1 - Baseline Characteristics of the Total Cohort	
	**Total Population, N (%)**
**Age, Mean (± SD)**	61.0 ± 15.4
**Gender**	
Male	690528 (88.7%)
Female	88210 (11.3%)
**Ethnicity**	
Non-Hispanic	687550 (88.3%)
Hispanic	65558 (8.42%)
Unknown	25630 (3.29%)
**Race**	
White	530508 (68.1%)
Black or African American	174892 (22.5%)
American Indian or Alaska Native	6299 (.809%)
Asian	8852 (1.14%)
Native Hawaiian or Other Pacific Islander	6930 (.890%)
Unknown	41907 (5.38%)
Missing	9350 (1.20%)
**BMI, Mean (± SD)**	30.1 ± 6.39
**CCI2yrs, Mean (± SD)**	1.95 ± 2.30

Table S2 - Incidence of Physical and Mental conditions between COVID-19 Negative vs. Positive Patients (Un-matched sample)

	Hospitalized Cohort					Outpatient Cohort			
	Total, n (%)	COVID-19 Negative, n (%)	COVID-19 Positive, n (%)	P Value		Total	COVID-19 Negative, n (%)	COVID-19 Positive, n (%)	P Value
**Pulmonary**									
Venous thromboembolism	4412 (3.24%)	3523 (2.91%)	889 (5.86%)	<.001		2573 (.426%)	1796 (.358%)	777 (.753%)	<.001
Pulmonary Circulation Disorders	4525 (3.33%)	3757 (3.11%)	768 (5.08%)	<.001		2290 (.377%)	1730 (.343%)	560 (.538%)	<.001
Sleep Apnea	2959 (3.11%)	2581 (3.03%)	378 (3.75%)	<.001		7543 (1.91%)	6517 (1.98%)	1026 (1.55%)	<.001
Chronic Lung Disease	4802 (5.29%)	3918 (4.88%)	884 (8.49%)	<.001		5720 (1.25%)	4716 (1.26%)	1004 (1.22%)	0.40
**Renal**									
Acute Kidney Injury	11790 (10.2%)	9705 (9.42%)	2085 (16.4%)	<.001		3939 (.673%)	3147 (.649%)	792 (.784%)	<.001
Chronic Kidney Disease	5350 (4.98%)	4591 (4.80%)	759 (6.48%)	<.001		3636 (.662%)	3061 (.673%)	575 (.613%)	0.04
Dialysis	1152 (.802%)	968 (.758%)	184 (1.15%)	<.001		621 (.100%)	515 (.100%)	106 (.100%)	0.98
**Cardiovascular**									
Ischemic Heart Disease	6763 (7.59%)	5986 (7.59%)	777 (7.60%)	0.97		5106 (1.04%)	4433 (1.09%)	673 (.788%)	<.001
Cerebrovascular Accident	3791 (2.97%)	3396 (2.99%)	395 (2.80%)	0.22		2557 (.435%)	2165 (.444%)	392 (.390%)	0.018
Congestive Heart Failure	7424 (6.82%)	6666 (6.90%)	758 (6.15%)	<.001		4175 (.737%)	3557 (.758%)	618 (.635%)	<.001
Peripheral Vascular Disease	3992 (3.60%)	3633 (3.70%)	359 (2.79%)	<.001		3322 (.594%)	2930 (.634%)	392 (.404%)	<.001
Cardiac Arrhythmia	9968 (11.5%)	8475 (11.1%)	1493 (15.1%)	<.001		8017 (1.67%)	6394 (1.62%)	1623 (1.94%)	<.001
Hypertension Uncomplicated	2837 (10.7%)	2484 (10.5%)	353 (12.2%)	0.004		4983 (2.11%)	4150 (2.13%)	833 (2.02%)	0.17

Hypertension Complicated	9411 (9.34%)	8077 (9.01%)	1334 (12.0%)	<.0001		4286 (.789%)	3628 (.807%)	658 (.702%)	<.001
**Endocrine**									
Diabetes Uncomplicated	2025 (2.45%)	1735 (2.34%)	290 (3.44%)	<.001		3225 (.734%)	2616 (.714%)	609 (.835%)	<.001
Diabetes Complicated	3052 (3.35%)	2562 (3.14%)	490 (5.16%)	<.001		3384 (.702%)	2809 (.701%)	575 (.710%)	0.78
**Others**									
Liver Disease	3238 (2.65%)	2876 (2.65%)	362 (2.61%)	0.75		3487 (.633%)	3040 (.668%)	447 (.466%)	<.001
Coagulopathy	4406 (3.27%)	3468 (2.90%)	938 (6.20%)	<.001		1867 (.309%)	1455 (.291%)	412 (.399%)	<.001
Fluid & Electrolytes Disorders	14197 (14.8%)	11546 (13.6%)	2651 (24.7%)	<.001		7072 (1.33%)	5740 (1.31%)	1332 (1.42%)	0.005
Neurological Disorders	5837 (4.90%)	4883 (4.60%)	954 (7.35%)	<.001		3708 (.661%)	3118 (.671%)	590 (.610%)	0.031
**Mental Disorders**									
Depressive Episode	5014 (5.06%)	4259 (4.84%)	755 (6.76%)	<.001		6934 (1.61%)	5889 (1.67%)	1045 (1.37%)	<.001
Panic Disorder	430 (.297%)	378 (.294%)	52 (.319%)	0.57		1165 (.192%)	1027 (.204%)	138 (.133%)	<.001
Generalized Anxiety	1066 (.779%)	954 (.786%)	112 (.723%)	0.40		3241 (.573%)	2768 (.591%)	473 (.487%)	<.001
PTSD	1535 (1.37%)	1361 (1.36%)	174 (1.39%)	0.79		4225 (.990%)	3639 (1.04%)	586 (.777%)	<.001
Adjustment Disorders	2566 (2.04%)	2204 (1.97%)	362 (2.58%)	<.001		5392 (1.02%)	4520 (1.03%)	872 (.957%)	0.040
Insomnia	4387 (3.84%)	3735 (3.68%)	652 (5.07%)	<.001		7591 (1.57%)	6509 (1.63%)	1082 (1.28%)	<.001
Dementia	3085 (2.28%)	2623 (2.17%)	462 (3.17%)	<.001		1700 (.280%)	1409 (.279%)	291 (.284%)	0.80

Figure S1: Likelihood of development of new physical and mental health conditions among matched COVID-19 positive hospitalized patients compared to matched COVID-19 positive outpatient.

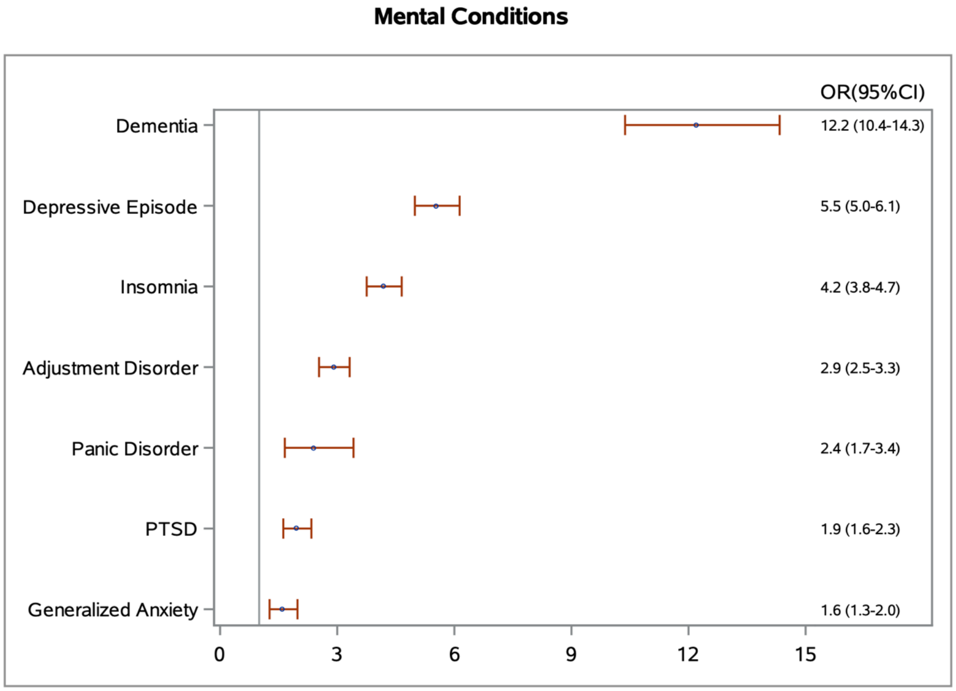

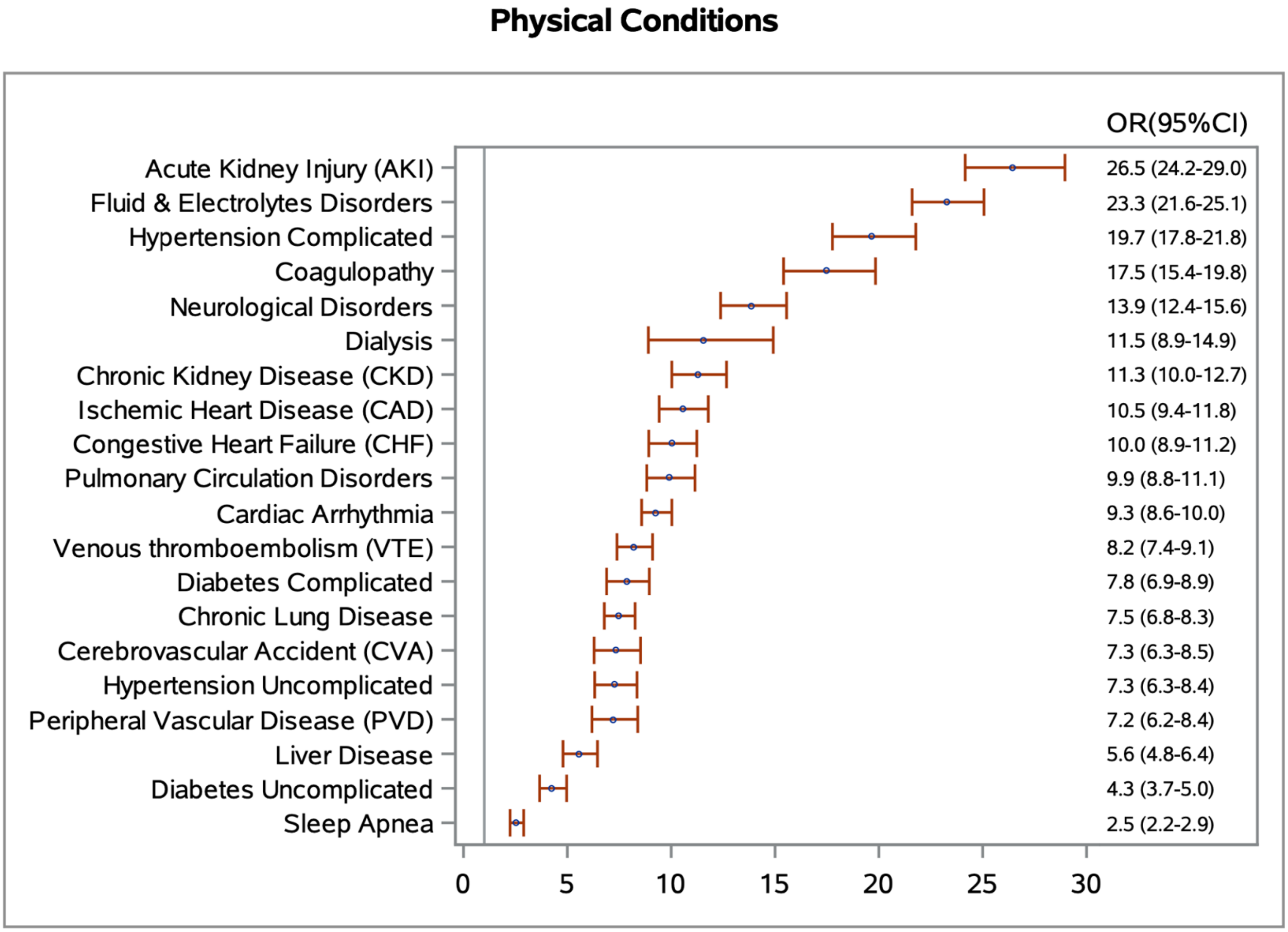

Table S3: Incidence of Physical and Mental conditions between matched COVID-19 Negative vs. Positive Patients (Follow up period – 15 days post-index to 3 months)

	Hospitalized Cohort				Outpatient Cohort		
	COVID-19 Negative, n (%)	COVID-19 Positive, n (%)	P Value		COVID-19 Negative, n (%)	COVID-19 Positive, n (%)	P Value
**Pulmonary**							
Venous thromboembolism	326 (2.43%)	665 (4.99%)	<.0001		265 (.283%)	640 (.681%)	<.0001
Pulmonary Circulation Disorders	306 (2.28%)	563 (4.24%)	<.0001		253 (.269%)	446 (.471%)	<.0001
Sleep Apnea	261 (2.88%)	264 (3.02%)	0.6005		1166 (2.01%)	849 (1.42%)	<.0001
Chronic Lung Disease	307 (3.26%)	546 (5.99%)	<.0001		671 (.926%)	749 (1.00%)	0.1290
**Renal**							
Acute Kidney Injury	744 (6.47%)	1171 (10.5%)	<.0001		362 (.397%)	455 (.495%)	0.0016
Chronic Kidney Disease	392 (3.69%)	466 (4.56%)	0.0016		436 (.507%)	436 (.511%)	0.9126
Dialysis	92 (.650%)	140 (.999%)	0.0012		67 (.069%)	90 (.093%)	0.0662
**Cardiovascular**							
Ischemic Heart Disease	543 (6.13%)	517 (5.79%)	0.3411		663 (.865%)	525 (.678%)	<.0001
Cerebrovascular Accident	263 (2.07%)	277 (2.23%)	0.3929		295 (.321%)	315 (.344%)	0.3849
Congestive Heart Failure	613 (5.66%)	528 (4.88%)	0.0104		550 (.625%)	510 (.576%)	0.1805
Peripheral Vascular Disease	323 (2.89%)	257 (2.28%)	0.0039		428 (.490%)	326 (.370%)	0.0001
Cardiac Arrhythmia	764 (8.98%)	1004 (11.6%)	<.0001		981 (1.32%)	1250 (1.64%)	<.0001
Hypertension Uncomplicated	220 (8.65%)	219 (8.94%)	0.7232		629 (1.71%)	651 (1.78%)	0.4811
Hypertension Complicated	604 (6.08%)	807 (8.27%)	<.0001		467 (.550%)	462 (.542%)	0.8225
**Endocrine**							
Diabetes Uncomplicated	161 (2.02%)	203 (2.78%)	0.0021		461 (.670%)	502 (.764%)	0.0411
Diabetes Complicated	235 (2.65%)	285 (3.45%)	0.0022		458 (.606%)	457 (.624%)	0.6601
**Others**							
Liver Disease	233 (1.90%)	228 (1.86%)	0.8004		486 (.563%)	363 (.416%)	<.0001
Coagulopathy	232 (1.73%)	556 (4.18%)	<.0001		170 (.182%)	254 (.270%)	<.0001
Fluid & Electrolytes Disorders	879 (9.02%)	1410 (14.9%)	<.0001		706 (.847%)	783 (.919%)	0.1139
Neurological Disorders	350 (2.92%)	657 (5.72%)	<.0001		444 (.508%)	420 (.476%)	0.3412
**Mental Disorders**							
Depressive Episode	331 (3.30%)	460 (4.60%)	<.0001		846 (1.26%)	749 (1.07%)	0.0010
Panic Disorder	24 (.168%)	32 (.223%)	0.2901		162 (.172%)	95 (.100%)	<.0001

Generalized Anxiety	64 (.471%)	79 (.578%)	0.2195		422 (.481%)	364 (.411%)	0.0273
PTSD	106 (.944%)	120 (1.08%)	0.2978		563 (.853%)	450 (.654%)	<.0001
Adjustment Disorder	187 (1.49%)	250 (2.00%)	0.0020		669 (.813%)	667 (.801%)	0.7935
Insomnia	301 (2.62%)	405 (3.57%)	<.0001		1005 (1.35%)	850 (1.10%)	<.0001
Dementia	208 (1.55%)	300 (2.32%)	<.0001		169 (.179%)	217 (.232%)	0.0106

Table S4: Incidence of Physical and Mental conditions between matched hospitalized COVID-19 positive vs. outpatient COVID-19 positive. (Follow up period – 15 days post-index to 3 months)

	Total, n (%)	Hospitalized COVID-19 Positive, n (%)	Outpatient COVID-19 Positive, n (%)	P Value
**Pulmonary**				
Venous thromboembolism	1305 (1.22%)	665 (4.99%)	640 (.681%)	<.0001
Pulmonary Circulation Disorders	1009 (.934%)	563 (4.24%)	446 (.471%)	<.0001
Sleep Apnea	1113 (1.62%)	264 (3.02%)	849 (1.42%)	<.0001
Chronic Lung Disease	1295 (1.55%)	546 (5.99%)	749 (1.00%)	<.0001
**Renal**				
Acute Kidney Injury	1626 (1.58%)	1171 (10.5%)	455 (.495%)	<.0001
Chronic Kidney Disease	902 (.944%)	466 (4.56%)	436 (.511%)	<.0001
Dialysis	230 (.208%)	140 (.999%)	90 (.093%)	<.0001
**Cardiovascular**				
Ischemic Heart Disease	1042 (1.21%)	517 (5.79%)	525 (.678%)	<.0001
Cerebrovascular Accident	592 (.570%)	277 (2.23%)	315 (.344%)	<.0001
Congestive Heart Failure	1038 (1.04%)	528 (4.88%)	510 (.576%)	<.0001
Peripheral Vascular Disease	583 (.586%)	257 (2.28%)	326 (.370%)	<.0001
Cardiac Arrhythmia	2254 (2.66%)	1004 (11.6%)	1250 (1.64%)	<.0001
Hypertension Uncomplicated	870 (2.23%)	219 (8.94%)	651 (1.78%)	<.0001
Hypertension Complicated	1269 (1.34%)	807 (8.27%)	462 (.542%)	<.0001
**Endocrine**				
Diabetes Uncomplicated	705 (.965%)	203 (2.78%)	502 (.764%)	<.0001
Diabetes Complicated	742 (.910%)	285 (3.45%)	457 (.624%)	<.0001
**Others**				
Liver Disease	591 (.594%)	228 (1.86%)	363 (.416%)	<.0001
Coagulopathy	810 (.754%)	556 (4.18%)	254 (.270%)	<.0001
Fluid & Electrolytes Disorders	2193 (2.32%)	1410 (14.9%)	783 (.919%)	<.0001
Neurological Disorders	1077 (1.08%)	657 (5.72%)	420 (.476%)	<.0001
**Mental Disorders**				
Depressive Episode	1209 (1.51%)	460 (4.60%)	749 (1.07%)	<.0001
Panic Disorder	127 (.117%)	32 (.223%)	95 (.100%)	<.0001
Generalized Anxiety	443 (.433%)	79 (.578%)	364 (.411%)	0.0055
PTSD	570 (.713%)	120 (1.08%)	450 (.654%)	<.0001
Adjustment Disorder	917 (.958%)	250 (2.00%)	667 (.801%)	<.0001
Insomnia	1255 (1.42%)	405 (3.57%)	850 (1.10%)	<.0001
Dementia	517 (.486%)	300 (2.32%)	217 (.232%)	<.0001

ICD-10 codes to define Physical and Mental Health Conditions

Pulmonary:
Venous Thromboembolism
I82, I26, I80.2, I80.1
Pulmonary Circulation Disorders
I26, I27, I28.0, I28.8, I28.9
Sleep Apnea
G47.3
Chronic Lung Disease
I27.8, I27.9, J40, J41, J42, J43, J44, J45, J46, J47, J60, J61, J62, J63, J64, J65, J66, J67, J68.4, J70.1, J70.3

Renal:
Acute Kidney Injury
N17
Chronic Kidney Disease (CKD)
I12.0, I13.1, N18, N19, N25.0, Z49.1, Z49.2, Z94.0, Z99.2, D63.1, E08.22, E09.22, E10.22, E11.22, E13.22, N99.0
Dialysis
R88.0, Z99.2, Z49.31, Z49.32, Z49.01, Z49.02, N18.6, Z91.15

Cardiovascular:
Ischemic Heart Disease or CAD
I20, I21, I22, I23, I24, I25

Cerebrovascular Accident

I60, I61, I62, I63, I66, I67, I68, I69
Congestive Heart Failure
I09.9, I11.0, I13.0, I13.2, I42.0, I4.5, I42.6, I42.7, I42.8, I42.9, I43, I50, P29.0
Peripheral Vascular Disease
I7.0, I71, I73.1, I73.8, I73.9, I77.1, I79.0, I79.2, K55.1, K55.8, K55.9, Z95.8, Z95.9
Cardiac Arrhythmia
I44.1, I44.2, I44.3, I45.6, I45.9, I47, I48, I49, R00.0, R00.1, R00.8, T82.1, Z45.0, Z95.0
Hypertension Uncomplicated
I10
Hypertension Complicated
I11, I12, I13, I15

Endocrine:
Diabetes Uncomplicated
E10.0, E10.1, E10.9, E11.0, E11.1, E11.9, E12.0, E12.1, E12.9, E13.0, E13.1, E13.9, E14.0, E14.1, E14.9

Diabetes Complicated
E10.2, E10.3, E10.4, E10.5, E10.6, E10.7, E10.8, E11.2, E11.3,E11.4, E11.5, E11.6, E11.7, E11.8, E12.2, E12.3, E12.4, E12.5, E12.6, E12.7, E12.8, E13.2, E13.3, E13.4, E13.5, E13.6, E13.7, E13.8, E14.2, E14.3, E14.4, E14.5, E14.6, E14.7, E14.8

Others:
Liver Disease
B18, I85, I86.4, I98.2, K70, K71.1, K71.3, K71.4, K71.5, K71.7, K72, K73, K74, K76.0, K76.2, K76.3, K76.4, K76.5, K76.6, K76.7, K76.8, K76.9, Z94.4
Coagulopathy

D65, D66, D67, D68, D69.1, D69.3, D69.4, D69.5, D69.6

Fluid & Electrolytes Disorders
E22.2, E86, E87

Neurological Disorders
G10, G11, G12, G13, G20, G21, G22, G25.4, G25.5, G31.2, G31.8, G31.9, G32, G35, G36, G37, G40, G41, G93.1, G93.4, R47.0, R56

Mental Disorders:

Depressive Episode
F32
Panic Disorder
F41.0
Generalized Anxiety
F41.1
PTSD
F43.1
Adjustment Disorders
F43.2
Insomnia
F51.0, G47.0
Dementia
F01, F02, Fo3, G30, G31.0, G31.83
